# Blood Prestin Levels in Normal Hearing and in Sensorineural Hearing Loss: A Scoping Review

**DOI:** 10.1097/AUD.0000000000001045

**Published:** 2021-04-22

**Authors:** Eleftheria Iliadou, Dimitrios Kikidis, Konstantinos Pastiadis, Christopher J. Plack, Athanasios Bibas

**Affiliations:** 1 1st University ENT Department, School of Medicine, National and Kapodistrian University of Athens, Athens, Greece; 2 School of Music Studies, Aristotle University of Thessaloniki, Thessaloniki, Greece; 3 Department of Psychology, Lancaster University, Lancaster, United Kingdom; 4 Manchester Centre for Audiology and Deafness, University of Manchester, Manchester Academic Health Science Centre, United Kingdom.

**Keywords:** Enzyme-linked immunosorbent assay (ELISA), Prestin, Biomarker, Sensorineural hearing loss

## Abstract

**Objectives:**

Recently, it has been hypothesized that blood prestin concentration levels may reflect cochlear damage and thus serve as an easily measurable, early sensorineural hearing loss (HL) biomarker. This is a scoping review aiming to identify and critically appraise current evidence on prestin blood levels and their temporal variation in rodents and humans with normal hearing and with sensorineural HL.

**Design:**

This study was designed and held according to PRISMA Extension for Scoping Reviews (PRISMA-ScR) guidelines. With no limitation with regards to study type, animal and human studies focusing on prestin blood levels in normal hearing and in sensorineural HL were sought in major databases such as Medline, Central Scopus, PROSPERO, and Clinicaltrials.gov. Results were then hand-searched. A data charting form was developed including the parameters of interest.

**Results:**

Seven studies focusing on measuring prestin blood levels by means of ELISA in rodents and human subjects with normal hearing and noise-induced, drug-induced, or idiopathic sudden HL were found eligible and were included in the analysis. According to these proof-of-concept studies, prestin can be detected in the circulation of subjects with no HL; however, normal ranges remain unclear. After cochlear damage, blood prestin levels seem to initially rise and then return to near or below baseline. The degree of their change relates with subjects’ degree of HL, damaged cochlear region and recovery. Prestin blood levels and their temporal variation seem to correlate with cochlear damage; however, methodological weaknesses, such as small sample size, lack of detailed phenotyping, insufficient exclusion of confounding factors, and short follow-up, do not allow for robust conclusions.

**Conclusions:**

Current findings support the value of studying blood prestin levels in normal hearing and HL and highlight a need for larger-scale longitudinal research.

## Abstract 2

Supplemental Digital Content is available in the text.

## INTRODUCTION

Biomarkers, or biological markers, are defined as patients’ characteristics that can be measured objectively, accurately, and reproducibly (Biomarkers Definitions Working Group, 2001). They serve as an indicator of normal biological processes, pathogenic processes, or pharmacologic responses to a therapeutic intervention (Strimbu & Tavel 2010). In the case of sensorineural hearing (SNHL), which accounts for the majority of hearing loss (HL) cases, they could identify early hearing impairment, potentially before it becomes measurable by standard audiometric procedures.

To date, no biomarker has been developed or validated in the case of SNHL. However, since the pathogenesis of many SNHL types occurs in a specific cell type in the inner ear, the outer hair cells (OHCs), these cells are suggested as a good target of future research and precision medicine (Kujawa & Liberman 2015; Eggermont 2017, 2019; Matsuoka et al. 2019). This type of cell is a main and early target of aging, various ototoxic substances, and overexposure to noise or acoustic trauma (Kujawa & Liberman 2015; Ryan et al. 2016). OHC loss or dysfunction may, in addition, have a pathogenetic role in idiopathic sudden SNHL (ISSHL) (Sun et al. 2020). Consequently, discovery of an OHC-specific biomarker and the assessment of the conditions under which it could help in the diagnosis and management of SNHL is of great priority.

Prestin is the fifth member of an 11-member membrane transporter superfamily (solute carrier family 26 or SLC26), which includes anion transporters and related proteins (Liberman et al. 2002; He et al. 2014). Prestin is exclusively produced in the cochlea. More specifically, it is situated in the lateral wall of OHCs and is responsible for their electromotility (Liberman et al. 2002; Parham 2015). Prestin’s exact role and regulation mechanisms have not been completely clarified (Mazurek et al. 2007; Xia et al. 2013; Matsunaga & Morimoto 2016). Its deficiency is associated with moderate SNHL. In mice, prestin gene deletion can cause loss of OHC electromotility in vitro and 40 to 60 dB loss of cochlear sensitivity in vivo, while heterozygotes present a 6 dB elevation of hearing thresholds (Liberman et al. 2002). Moreover, in vitro OHC damage due to ototoxic substances and high-intensity noise increases the expression rate of the responsible gene (Mazurek et al. 2007; Xia et al. 2013). To date, there is only one observational study assessing the auditory results of prestin gene mutation in humans (two identical twins); its results also imply a sensorineural loss of about 40 to 60 dB (Matsunaga & Morimoto 2016).

Apart from cochlear prestin, circulating prestin has also been observed in the blood of animals and humans with or without HL (Parham 2015; Dogan et al. 2018; Tovi et al. 2019). Its presence in the blood has been detected by means of enzyme-linked immunosorbent assay (ELISA) and could be explained either by its small size (80 kDa), which may allow crossing of the blood-labyrinthine barrier or by its engulfment by phagosomes after OHC apoptosis. ELISA is capable of detecting blood prestin even in small quantities, where less than 1% of OHCs are lost (long before audiological symptoms or abnormal audiometric outcomes appear) (Parham 2015).

All the above arguments have led to the hypothesis that prestin blood levels could reflect changes or damage in the cochlea, and more specifically in the OHCs, and thus serve as an easily measurable, early SNHL biomarker (Parham 2015). Consequently, evaluating potential changes in prestin blood concentration in patients suffering from SNHL has attracted researchers’ interest. A scoping review of all the available scientific evidence could help the design and execution of further research in this particular domain. To the best of our knowledge, no such review has been conducted to date.

## MATERIALS AND METHODS

### Objectives

This is a scoping review of current clinical and basic science literature on the measurement of prestin levels in the blood of normal and hearing-impaired animals and human subjects, aiming at systematically mapping the research conducted in this area. Identification of the limitations of previous works, methodological pitfalls, or gaps in current knowledge are a prerequisite to understand under which conditions prestin blood levels can have a meaningful interpretation.

The following research question was formulated: What is known from the literature about prestin blood levels and its temporal variations in people and animals with or without SNHL?

### Methods

The protocol of this study was drafted according to PRISMA Extension for Scoping Reviews (PRISMA-ScR) guidelines (Shamseer et al. 2015; Tricco et al. 2018).

### Eligibility Criteria

Population: humans and animals with or without SNHL
Intervention: measurement of prestin blood levels
Comparator: not applicable
Outcome: prestin blood levels in healthy controls and hearing-impaired, temporal variation of prestin blood levels, correlation of prestin blood levels with HL
Inclusion Criteria: controlled experimental studies [controlled clinical trials and randomized controlled trials], observational studies [longitudinal and cross-sectional studies], reviews. Publication type and language: English, French, Spanish, or German-language journal articles. Publication year: last 10 years. Particulars: There was no restriction in types of SNHL. Sudden HL, noise trauma, hereditary HL, and so on, were all included in the review. Both human and non-human studies have been included.
Exclusion Criteria: Studies in languages other than the aforementioned ones. No full text available.

### Information Sources

Major databases of Medline, Central, and Scopus were searched for eligible studies by two reviewers independently. The gray literature was sought in PROSPERO, Clinicaltrials.gov, EU Clinical Trials Register, and the lists of abstracts in major Audiology- and Otoneurology-related conferences of the past 6 years. The results were then hand-searched (Hopewell et al. 2002).

### Search

The Medline search was conducted via Pubmed by using free text and MeSH terms. Predefined search strategies and selection criteria were used to evaluate the eligibility of studies. Final syntax follows:

(prestin) AND ((hearing loss) OR (hearing impairment) OR “Hearing Loss” [Mesh] OR “Hearing Loss, Sensorineural” [Mesh] OR “Hearing Loss, Noise-Induced” [Mesh] OR “Hearing Loss, Sudden” [Mesh] OR “Deafness” [Mesh])

Adding a third search term such as “ELISA,” “Enzyme-Linked Immunosorbent Assay,” [Mesh] or “antibodies” was finally rejected since a number of studies were omitted. The NOT Boolean Operator was tried in an effort to exclude conductive-HL-focused studies, but it was finally rejected since it did not change the number of results.

Search in the other aforementioned databases followed the same principles, using keywords and MeSH terms wherever available. Because this review is focusing only on studies measuring prestin in the blood and this has been introduced as a procedure only recently (Parham et al. 2014), our research was limited to the last 10 years.

### Selection of Sources of Evidence

All studies were screened, first by title and abstract and subsequently by full text to identify and exclude those that were irrelevant, duplicates, or in other than the approved language.

### Data Charting

A data charting form was developed including the parameters of interest for the particular study.

### Data Items

Data about article identification (author, journal, year of publication), article characteristics (e.g., country of origin, language, funding), population characteristics (human or other species, age, type of HL), prestin level measurement (methodology, setting, results) were extracted from the included studies. A comprehensive summary will be presented in Results (Section 3.2).

### Synthesis of Results

We present the included studies and summarize the type of settings, populations, and study designs, with emphasis on our predefined scientific queries (study’s timeline, results in prestin blood level as measured in controls and hearing-impaired subjects).

## RESULTS

### Selection of Sources of Evidence

**Fig. 1.**
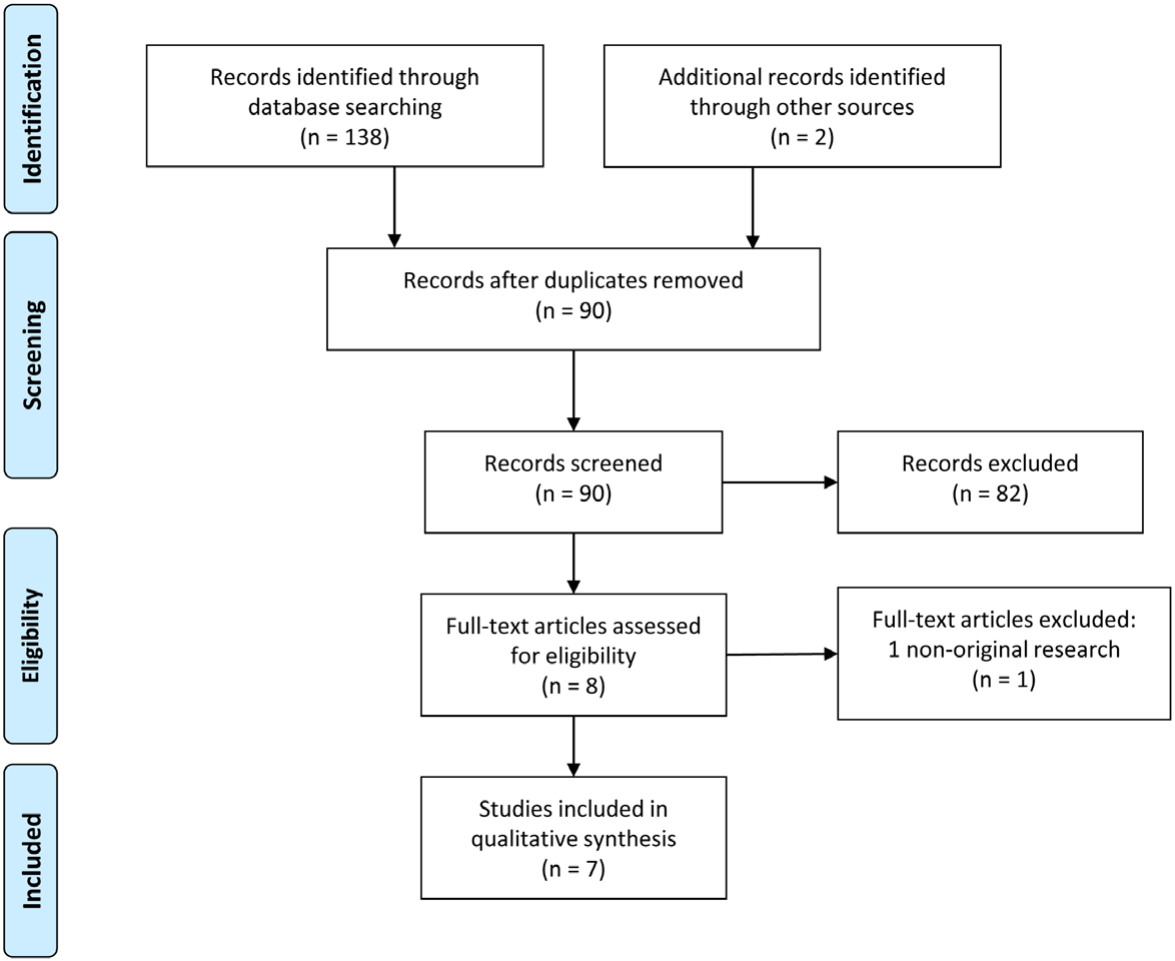
Selection of sources of evidence. Seven studies fulfilled the predefined inclusion criteria.

Procedures followed for the selection of included studies are presented in Figure 1.

### Characteristics of Sources of Evidence

Article identification information (author, year, and journal of publication) and comprehensive comments on the methodology and results of each one are included in Table 1 and Table in Supplemental Digital Content 1, http://links.lww.com/EANDH/A798.

**TABLE 1.** Characteristics of included human studies

Author, yr, journal	Title	Population	Summary	Audiometric assessment	Findings
Sun et al. *Ear, Nose and Throat Journal* 2019	A Preliminary Report on the Investigation of Prestin as a Biomarker for Idiopathic Sudden Sensorineural Hearing Loss.	Adults with unilateral ISSHL [n = 14 (8 females), age = 57.9 (15.4), mean (SD)]	Measurement of prestin blood levels in 14 patients hospitalized for ISSHL, before and after treatment with oral prednisolone 1 mg/kg for 5 days, IV Gingko biloba, intramuscular cobamamide (±batroxobin) and comparison with 24 adults with no history of hearing loss (age/sex-matched control group)	Pure-tone audiometry - Arithmetic mean value of 0.5, 1, 2, and 4 kHz - Recovery according to Siegel’s criteria (nominal)	- Prestin was detected in all participants (patients/controls) - Prestin levels in ISSHL patients before treatment were significantly higher compared with control patients - Approximately half of the patients with ISSHL had higher prestin concentrations than the average level of the control group - Plasma prestin levels before treatment in patients with ISSHL did not correlate with treatment outcomes
Hana and Bawi *Ibnosina Journal of Medicine and Biomedical Sciences* 2018	Prestin, otolin-1 Regulation, and Human 8.oxoG DNA Glycosylase 1 Gene Polymorphisms in Noise-Induced Hearing Loss	Adults with occupational (NIHL). [n = 300, age = 40.5 (5.2), mean (SD), M:F = 93.2%]	Measurement of prestin blood levels in 300 patients with NIHL in comparison with 200 workers (age, sex, and occupational noise exposure-matched) with normal hearing	Pure-tone audiometry - 0.5, 1, 2, 4, 6 kHz - Nominal according to degree of hearing loss	- Prestin was detected in all participants (patients/controls) - Significant positive correlations were detected between prestin level and the severity of NIHL, otolin-1 level, 8–OxoG, and Cys/Cys genotype

All prestin blood level measurements were conducted by means of ELISA.

SD, standard deviation; ISSHL, idiopathic sensorineural hearing loss; IV, intravenous; NIHL, noise-induced hearing loss.

### Prestin Blood Levels Without Hearing Loss or Noise Trauma

Four out of seven included studies evaluated the levels of prestin in the blood of human subjects or animals with no HL or exposure to noise or to ototoxic agents. In a recent clinical study by Sun et al. (2020), 24 people (13 females) referred as of normal-hearing capacity were age- and sex-matched to 14 (eight females) ISSHL patients. In this control group, which had no HL history, prestin levels ranged from 85.4 to 1628.25 pg/ml, with an average of 840.24 (±496.22) pg/ml [mean (SD)]. Mean age was higher than 54 years; however, no further information on the control group’s characteristics was available (Table 1).

A larger-scale cross-sectional study by Hana and Bawi (2018), assessing prestin blood levels in 300 workers with noise-induced hearing loss (NIHL), also showed that prestin was present in the blood of 200 volunteers that served as controls [100.9 (±16.7) pg/ml, mean (SD)] (Table 2). The mean age of the control group was estimated at 40.3 (±3.9) years. Their occupational exposure to noise was measured using a sound level meter at their workplace and reported according to the duration of occupational noise exposure in months [18.2 (±7.4) mo, mean (SD)] and exposure levels [87.0 (±7.6) dBA, mean (SD)] separately. Overall exposure was unclear since recreational exposure was not reported. The earlier information allowed the authors to match the control to the patient group by age, gender, and occupational exposure. The authors also asked patients and controls for a history of ototoxic drugs usage, hearing-related family history, and smoking, although they only controlled for smoking in the analysis.

**TABLE 2.** Mean prestin concentration levels in two human studies

	Patients	Controls	*p* ^†^
Hana and Bawi 2018	300 workers with NIHL	200 workers with normal hearing	
Prestin level in pg/ml (SD), before treatment	169 (88.4)	100.9 (16.7)	0.04*
Prestin level in pg/ml (SD), 1 mo after treatment	114 (99.2)	–	0.04*
Sun et al. 2019	14 patients with ISSHL	24 with normal hearing	
Prestin level in pg/ml (SD), before treatment	1955.98 (2501.48)	840.24 (496.22)	<0.01*
Prestin level in pg/ml (SD), after treatment	1653.26 (1967.60)	–	0.06

*Significant at an alpha of 0.05.

^†^*p* value for comparison between patients and controls.

SD, standard deviation; ISSHL, idiopathic sudden sensorineural hearing loss; NIHL, noise-induced hearing loss.

Animal studies have also shown that prestin can be detected in controls’ blood. Parham et al. (2019) observed that prestin levels ranged from 125 to 245.7 pg/ml [177.9 (±4.3) pg/ml, mean (SD)] in 46 male Wistar rats with no prior exposure to ototoxic noise or drugs. In another male Wistar rat model, measuring prestin levels after exposure to ototoxic factors (amikacin, cisplatin), Dogan et al. (2018) also showed that prestin could be detected in the blood of the control, ototoxic-drug-free, group [n = 10, 377.0 (±135.3) pg/ml, mean (SD)].

### Relation of Blood Prestin Levels to Hearing Loss

All included studies were focused on SNHL. Sun et al. (2020) included patients hospitalized with ISSHL. Hana and Bawi (2018) evaluated prestin blood levels in patients with NIHL. Animal studies included rats and guinea pigs that were exposed to ototoxic substances, such as aminoglycosides and cisplatin (Liba et al. 2017; Dogan et al. 2018; Naples et al. 2018), and thus present SNHL due to ototoxicity; or to hazardous levels of noise, and thus present NIHL (Parham & Dyhrfjeld-Johnsen 2016; Parham et al. 2019).

In the ISSHL study of Sun et al. (2020), 14 participants with mean age 57.9 (±15.4) years [mean (SD)] presented significantly higher levels of prestin in their blood before treatment compared with controls (*p* < 0.001, statistical test not reported). All measurements before treatment were conducted within 7 days from the onset of HL and ranged from 190.30 to 9648.80 pg/ml, with a 1955.98 (±2501.48) pg/ml [mean (SD)] average concentration. Half of ISSHL patients presented higher concentration levels compared with the average value for the control group and 35.7% of ISSHL participants had higher prestin levels than the highest value detected in the control participants. Measurements were repeated at the end of treatment, within 4 to 11 days after the initial measurement, and ranged from 0 to 7610.45 pg/ml, with an average concentration of 1653.26 (±1967.60) pg/ml [mean (SD)]. Prestin blood concentration before treatment did not correlate with treatment outcomes (Table 2). Six out of 10 participants who recovered from ISSHL had decreased blood prestin. All four participants that did not recover presented increased prestin levels.

In the human NIHL study, Hana and Bawi (2018) revealed a significant difference in prestin blood levels between the patient and control group immediately after noise exposure (Table 2). In comparison to controls, NIHL patients’ age, gender ratio, smoking habits, and occupational exposure to noise [18.6 (±7.6) mo, mean (SD) and 87.0 (±7.6) dBA, mean (SD)] did not differ significantly. One month after treatment (no information on type of treatment was provided), mean prestin concentrations in the NIHL group were 55% lower than that initially observed [114 (±99.2) pg/ml, mean (SD)]. These values differed significantly from the ones before treatment (t = 4.3, *p* = 0.02) and from the control group (Table 2). Significant positive correlations were reported between prestin level and severity of HL (r = 0.971), otolin-1 level (r = 0.776), 8–OhdG (r = 0.556), and Cys/Cys genotype (r = 0.828).

The effect of the noxious agent (noise or drug) in rodent models has been verified in all relevant studies by means of histological and audiometric testing [auditory brainstem responses (ABR) and distortion product otoacoustic emissions (DPOAEs)]. Liba et al. (2017) observed an increase in blood concentrations of prestin both in guinea pigs that had an increase in ABR thresholds and in mice that were found resistant to cisplatin according to their audiometric evaluation.

Dogan et al. (2018) exposed rats to low and high doses of amikacin (200 and 600 mg/kg/day, respectively) for 10 days and cisplatin (single dose of 5 and 15 mg/kg, respectively) for 3 days and conducted prestin measurements immediately after the end of the experiment. They report that their audiometric findings via DPOAEs showed significant changes at specific frequencies (4, 6, and 8 kHz). Mean prestin blood levels were found to be 411.3 (±73.1) pg/ml [mean (SD)] in the low-dose amikacin group and 512.6 (±106.0) pg/ml [mean (SD)] in the high-dose amikacin group. Corresponding values for cisplatin were 455.0 (±74.2) pg/ml [mean (SD)] in the low-dose group and 555.3 (±47.9) pg/ml [mean (SD)] in the high-dose group (Dogan et al. 2018). Significant differences were found in blood prestin between the low and high amikacin groups, between the low and high cisplatin groups, and for all treatment, groups compared with controls [377.0 (±135.3) pg/ml, mean(SD)]. Prestin blood levels were significantly correlated with the threshold changes in those frequencies where a significant threshold shift was detected in the DPOAEs. In the study of Parham and Dyhrfjeld-Johnsen (2016), blood prestin concentration showed a linear negative relationship with DPOAE level change (r = 0.563, *p* = 0.01) and a linear positive relationship with ABR threshold change (r = 0.46, *p* = 0.036) at 14 days after exposure to noise. Naples et al. (2018) found that the increase in ABR threshold was recovered at day 7 and 14 in the guinea pigs that received diltiazem as otoprotectant after cisplatin exposure. Prestin blood concentrations were in accordance with these functional results, and no significant change in prestin levels was observed in the diltiazem group.

Parham et al. (2019) included histological testing, ABR and DPOAEs to the assessment of cochlear damage due to exposure to 110 dB (low noise group) and 120 dB SPL (high noise group). In this particular study, agreement was observed between functional, histological, and serological findings: significantly greater loss of OHCs was observed in the 120 dB SPL group and was associated with a greater extent of functional changes and decrease in prestin levels compared with the 110 dB SPL group.

### Variation in Prestin Blood Levels Over Time After Cochlear Damage

In the recent animal study by Parham et al. (2019), prestin levels were measured at 4 hr, 24 hr, 48 hr, 72 hr, 7 and 14 days after 2 hr of exposure to noise of 110 and 120 dB SPL. The study found a noise-level-dependent change of prestin over time; after an initial peak of prestin concentration right after the noise trauma (4 hr), the overall (14 days) statistically significant decrease of prestin concentration in comparison to preexposure values was found to be less than 5% for the low-dose group (approximately 10 pg/ml) and more than 10% for the “loud” group (approximately 30 pg/ml). Two additional animal studies provide information on the change of prestin levels over time; the first study by Parham and Dyhrfjeld-Johnsen (2016) evaluated blood prestin 14 days after noise exposure (rats). The second one by Liba et al. (2017) at 1, 3, 7, 14 days after one single dose of cisplatin at 8 mg/kg (rats and guinea pigs). The first study found that blood prestin concentrations in noise-exposed rats were significantly below control levels at day 14 after the noise trauma (Parham et al. 2019). The second study reports that prestin rose to a maximum value on day 7 (mice) and day 3 (guinea pigs) after cisplatin treatment and then declined back to or below baseline/control levels on day 14 (Liba et al. 2017).

Naples et al. (2018) explored prestin blood level changes over time after exposure to ototoxic substances. Prestin levels were measured at 1, 2, 3, 7, and 14 days postcisplatin administration in 20 guinea pigs. Ten of them received treatment with diltiazem (as otoprotection), while 10 received saline and served as controls. In this particular study, the diltiazem group had no significant change of prestin levels before and after cisplatin. In the control group, the rise from baseline values reached a maximum at day 2 postcisplatin administration and remained elevated at day 3 before trending back toward baseline at days 7 and 14. The mean percentage changes in prestin level for days 1 to 3 were statistically significant compared with baseline.

**Fig. 2.**
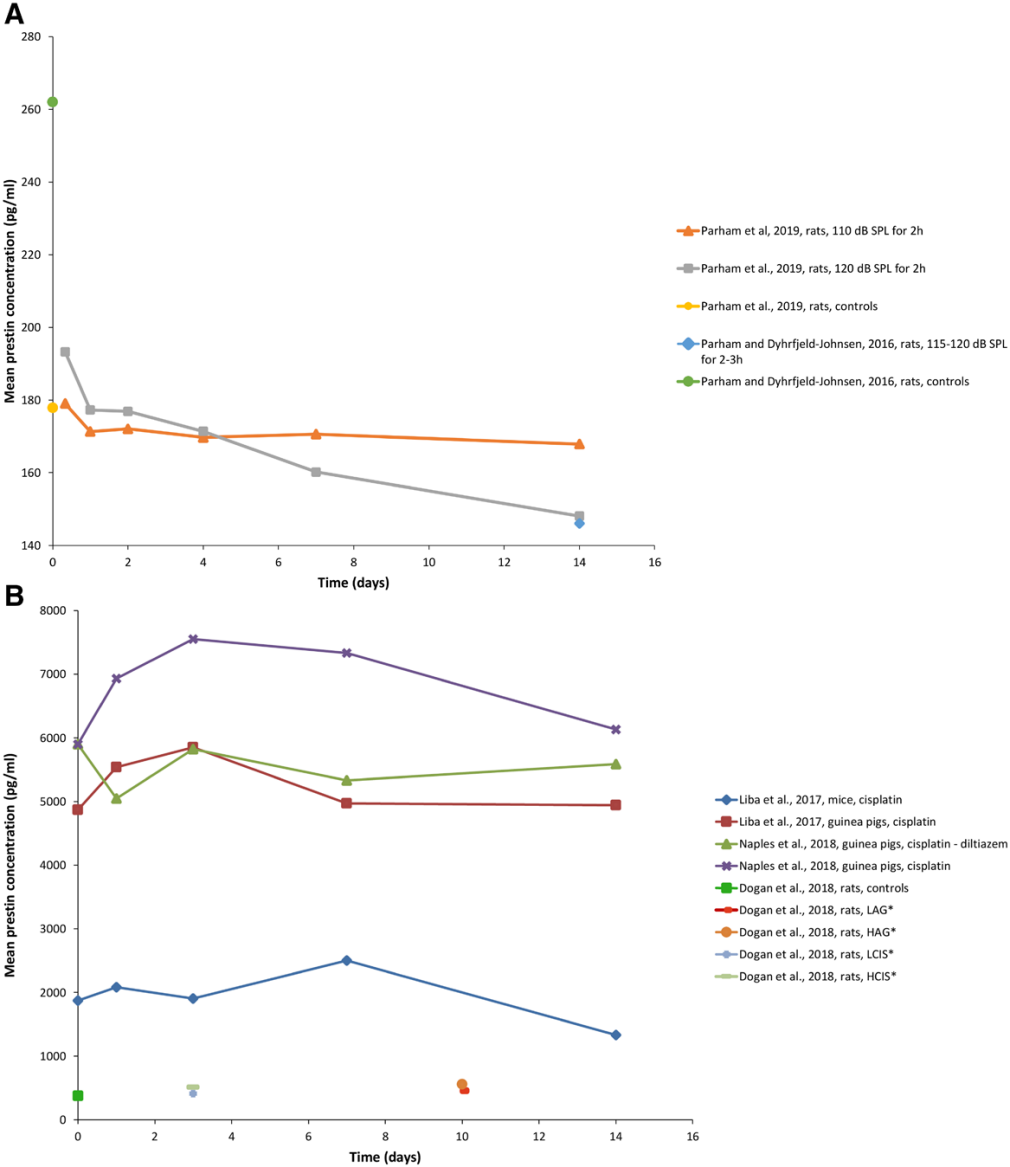
Average prestin concentrations as a function of time after trauma in the two NIHL rodent models over time (A) and in the three-drug ototoxicity rodent models (B). (Data extracted from the original graphs via WebPlotDigitizer). *HAG = 600 mg/kg/day of amikacin (10 days), LAG = 200 mg/kg/day of amikacin (10 days), LCIS = 5 mg/kg of cisplatin (3 days), HCIS = 15 mg/kg of cisplatin (3 days).

A summary of the results of prestin concentration after trauma is provided in Figure 2. It should be noted that no specification of the time of day or point on circadian cycle was mentioned in any of the included studies.

## DISCUSSION

It has been hypothesized recently that, apart from the changes due to normal prestin turnover within OHCs, blood prestin concentrations may also be related to cochlear damage (Parham 2015). Building on this idea, prestin blood levels have been measured in several rodent and human studies with NIHL, ISSHL, or drug-induced HL. The rationale for these studies is that these types of SNHL seem to be associated with OHC damage (Ryan et al. 2016; Tovi et al. 2019). ELISA is the only blood prestin detection method that has been identified in the present review (Table 1 and Table in Supplemental Digital Content 1, http://links.lww.com/EANDH/A798). A new electrochemical immuno-biosensor for circulating biomarkers of the inner ear (otolin-1 and prestin) has been recently proposed (Mahshid & Dabdoub 2020). Nevertheless, no data from rodent or human studies are yet available, and further research is needed before validating the conditions under which this method can be used in research and clinical practice.

An *increase* in blood prestin concentrations in the case of OHC damage or loss may be explained by the release of prestin from the OHCs directly through the blood-labyrinth barrier or by means of phagosomes in the supporting cells (in the short term) and the functional up-regulation of prestin expression in the residual OHCs (longer term) (Abrashkin et al. 2006; Liba et al. 2017). Although there is controversy concerning the correlation of prestin mRNA levels to prestin protein levels (Cheatham et al. 2005; Liberman et al. 2002), data from 5- to 6-week-old wild-type CBA/CaJ mice show that prestin has been up-regulated by 32% to 58% within remaining OHCs after noise exposure (Xia et al. 2013). Similarly, in 5-week-old wild-type, CBA/CaJ mice with diphtheria toxin-induced SNHL (intraperitoneal injections of diphtheria toxin, 50 ng/g for 3 days in a row) prestin up-regulation seems to be locally regulated by the steady state transducer bias current with no involvement of centrally mediated efferent feedback (Roux et al. 2006; Song et al. 2015). Based on current evidence, it is difficult to conclude whether the increase of prestin blood concentration reflects cochlear damage directly, via passage of free prestin molecules to the circulation, or a cochlear compensation mechanism for temporarily or permanently damaged hair cells through up-regulation of the prestin gene in the remaining OHCs. It is also unclear how any up-regulation of the prestin gene may reflect to inner ear prestin levels or to prestin’s blood concentration. Better understanding of the pathophysiological mechanisms that are involved in noise-induced cochlear damage and prestin up-regulation may clarify the source of the postnoise exposure increase of prestin blood concentration observed in the studies mentioned in this review.

A *decrease* in blood prestin concentration in the case of OHC damage or loss may be the product of a dynamic equilibrium of cochlear function where the remaining, fewer OHCs release less prestin into circulation (Parham et al. 2019). Decreased blood prestin may also be a consequence of the disruption of the balance between the production of free radicals and the antioxidant defense system in the cochlea that can occur after exposure to intense noise or other noxious agents. Hana and Bawi (2018) observed a significant positive correlation between prestin blood levels and blood 8 OHdg. Nevertheless, evidence from age- and noise-related HL rodent models shows that intracochlear reactive oxidative species accumulation may affect, to some degree, OHCs’ lateral wall and electromotility (Chen 2006; Chen et al. 2009; Lee et al. 2019). OHC structure changes may decrease prestin’s cellular, and thus free intracochlear, concentration, which may consequently have an effect on its blood concentration. Intracochlear reactive oxidative species may lead to oxidation of different elements of the prestin molecule or to the formation of protein–protein cross-linkages (Berlett & Stadtman 1997; Chen 2006).

Over the studies reviewed here, a short-term increase in prestin blood concentration and a long-term decrease below baseline has been observed following trauma. This finding agrees with previous studies on prestin gene regulation. Prestin up-regulation has been shown in rats with verified HL (functional and histological assessment) after exposure to noise (10 to 20 kHz, 110 dB SPL for 4 hr). In this animal model, prestin expression peaked at third postexposure day (4.9 ± 0.3 folds of increase) and returned progressively to baseline 4 weeks after noise exposure (Chen 2006). Similarly, in another rodent model, after monaural noise exposure, change of endocochlear prestin mRNA levels was associated with the degree of HL and differed among different parts of the cochlea increasing with a base-to-apex gradient (Mazurek et al. 2007). At first, exposed rats and guinea pigs presented moderate NIHL (15 to 25 dB DPOAE threshold shift), and prestin mRNA increased. One-week postexposure, NIHL severity had increased (by about 30 dB) and prestin blood levels tended to decline. It is interesting that DPOAE decrease and prestin up-regulation was observed in the contralateral ear (nonexposed), as well (Mazurek et al. 2007).

Long-term prestin regulation in the case of SNHL remains unclear. Immunohistochemical staining of the cochlea of F344 rats with age-related HL has indicated that prestin is reduced and that this age-related reduction may precede hair cell degeneration (Chen et al. 2009). Nevertheless, data concerning human prestin regulation and blood concentration are still missing.

### Prestin Blood Levels Without Hearing Loss or Noise Trauma

It has been hypothesized that prestin is detected in the blood of “naïve” rodents due to its normal turnover in the OHC membrane (Parham & Dyhrfjeld-Johnsen 2016). This may further imply that normal values differ per species and are correlated with the number of OHCs and the length of the cochlea (Liba et al. 2017). In human studies, prestin was found in the blood of people identified as of “normal hearing” (Hana & Bawi 2018; Sun et al. 2020). However, there is a lack of information concerning their medical and noise exposure history and their full audiometric profile. This information is necessary to confirm that their hearing status was indeed healthy. Additional data on prestin levels in people of different ages, who would have a full history and audiometric profiling, would be extremely useful in developing norms and determining which prestin levels are part of the normal physiological turnover of OHCs and which indicate cochlear damage.

### Relation of Plasma Prestin Levels to Hearing Loss

Prestin blood concentration changed significantly in rodents with acquired HL when compared with baseline measurements or controls (Parham & Dyhrfjeld-Johnsen 2016; Dogan et al. 2018; Parham et al. 2019). Using nonexposed “naïve” rodents and assessing the effect of noise or ototoxic agents on their hearing by means of histological and functional assessment allows the safe correlation of each change in prestin levels with specific phase of cochlear damage.

Previous studies on intracochlear prestin expression indicate its base-to-apex gradient increase and its association with the degree of HL (Chen 2006; Mazurek et al. 2007). Similarly, blood prestin levels show an association to the cause and degree of cochlear damage. In the case of NIHL, prestin levels have been associated with the levels of noise exposure and the degree of temporary and permanent threshold shift that has been caused. In the study of Parham et al. (2019), 20 rats exposed to intense octave band noise at 120 dB SPL showed significant changes in prestin concentration when compared with the changes observed in the 110 dB SPL exposed group. In the case of ototoxicity, groups with high doses of cisplatin and amikacin presented both higher degrees of HL and prestin blood concentration (Dogan et al. 2018).

A very interesting finding is that, in specific cases, prestin expression change precedes auditory findings and may have a higher predictive value than audiometric assessment. Liba et al. (2017) observed a rise in blood prestin levels at day 2 postcisplatin administration. This rise preceded the onset of significant ABR changes. This observation may be explained by the fact that early up-regulation of intracochlear prestin may maintain normal cochlear function. The findings of Parham et al. (2019) on NIHL suggest that an early rise of blood prestin is a better prognostic marker than ABR or DPOAEs threshold shifts. Nevertheless, it is not clear if, and under which conditions, prestin blood concentration is indeed more sensitive than standard audiometric testing. Further research, combining histological examination of the cochlea, DNA expression determination, functional auditory testing, and ELISA could clarify better the correlations and time sequencing among cochlear trauma, OHC loss, prestin gene expression, prestin protein endocochlear/blood concentration, and auditory function.

In the human ISSHL study, Sun et al. (2020) found a significant difference in prestin blood levels between patients and controls (Sun et al. 2020). However, only half of the ISSHL participants had higher levels of prestin blood concentration when compared with controls. According to the authors, this finding suggests that only some of the ISSHL patients present true OHC damage and that prestin could serve as a means of their identification. However, before being able to generalize this claim, larger-scale data on hearing phenotypes of ISSHL patients and controls are needed. Prestin concentration has shown some association with the degree of recovery, but more data are needed before being able to generalize this finding. In the NIHL study by Hana and Bawi, patients’ prestin was significantly greater, both before and after treatment (1 mo later) when compared with controls (Hana & Bawi 2018). However, as mentioned before, methodological issues of those studies (small sample size, incomplete audiometric profiling, and unclear timeline of measurements) do not allow their results to be generalized easily.

### Variation in Prestin Blood Levels Over Time After Cochlear Damage

Recent evidence implies that there is a circadian regulation of auditory function and noise sensitivity (Basinou et al. 2017). Quantitative real-time polymerase chain reaction has revealed circadian regulation of various endocochlear transcripts, while specific neurotrophic factors that are associated with cochlear neurogenesis and homeostasis, such as *brain-derived neurotrophic factor*, have also shown a circadian pattern (Singer et al. 2014). Current evidence from rodent and human studies does not clarify the effect of circadian regulation on prestin blood levels.

Preliminary data from animal and human studies have shown that prestin blood levels depend on the interval between the exposure to the ototoxic agent (drug or noise) and its measurement by means of ELISA. Two rodent models have shown that prestin presents an increase in the blood immediately after exposure to noise and then returns to baseline, or below baseline values, 14 days after (Parham & Dyhrfjeld-Johnsen 2016; Parham et al. 2019). No data later than 14 days after trauma are available to date. No information on the specific time of the day that the ototoxic agent was applied or the blood was drawn is available either.

To date, no human studies have assessed prestin blood level variation over time after exposure to noise or to an ototoxic agent in the absence of potential confounding factors after the initial time point, such as continuation of the exposure to the noxious agent (noise or ototoxic drug) or participants’ therapeutic treatment for HL. To assess the true change of blood prestin after cochlear damage, factors that may potentially affect its intracochlear regulation and concentration should be avoided after baseline measurement, and all variables but time should be held constant. Consequently, longitudinal human studies focusing on prestin concentration over time are warranted.

In the two human models included in this review, prestin was measured before and after treatment for NIHL and ISSHL (Hana & Bawi 2018; Sun et al. 2020). However, no specific timeline of participants’ HL or the relation between day of onset of the HL or HL diagnosis and the day of prestin measurement was provided. No information on the specific time of the day that participants were exposed to noise or that the blood was drawn is available either.

It should be noted that if prestin levels return to near-normal shortly after trauma, then they may be of little use in the diagnosis of established HL. Further research with multiple measurements during day- and nighttime, for different hearing phenotypes, with and without exposure to noxious agents (e.g., noise), is needed to evaluate this. Long-term prestin gene functional expression and prestin protein release into regulation also needs further understanding.

### Methodological Pitfalls

Over the human studies reviewed here, there is great heterogeneity in primary endpoints, such as the mean prestin concentration levels and their range both in control and patient groups. There is also a lack of detail on important methodological issues. These differences could explain the heterogeneous results.

Age is another parameter that may be associated with the variability of prestin blood levels. Data from rodent models show that prestin levels in OHCs, evaluated semi-quantitatively by immunohistochemical staining, are reduced in the aging cochlea of F344 rats (Chen et al. 2009).

With regards to sample characteristics, other parameters may also affect prestin concentration levels and should be better clarified before drawing any conclusions. Different causes of HL are linked to different pathophysiological mechanisms and possibly different endocochlear and blood behavior. In a recent human study focused on ISSHL, Tovi et al. (2018) state that ELISA detected antiprestin antibodies in the serum of only two out of 63 patients with unilateral ISSHL. These findings, along with the fact that ISSHL pathophysiology remains unclear and differs from NIHL, make comparison of the two human studies reviewed here challenging (Greco et al. 2011).

Duration of the symptoms, age of onset, severity of HL, and interval between treatment and onset of HL, are all considered as SNHL prognostic factors. In the NIHL study by Hana and Bawi, it is described that patients were referred to the hospital because of NIHL, received treatment, and were tested for prestin blood levels before and after this treatment. It is mentioned that tinnitus and HL were the main symptoms. However, information is lacking on whether acoustic trauma was acute or chronic and on the specific kind of treatment patients have undergone. This information is important since different pathophysiological mechanisms could be involved (Le et al. 2017). Concerning the audiometric profile of patients, they have been classified according to their degree of HL as it was defined by average threshold at 0.5, 1, 2, 3, 4, 6 kHz in the pure-tone audiogram. No further audiometric evaluation was conducted (Hana & Bawi 2018). Similarly, in the ISSHL study of Sun et al., the prestin concentrations of 14 participants were included in the analysis, without any detail on their clinical variables being taken into account.

Hana and Bawi (2018) matched the two groups by age, gender, and occupational exposure. They asked patients and controls for a history of ototoxic drug usage and hearing-related family history, but they did not control for these in the analysis. They also included questions about work and disease history; however, this information is not reported in the article. Participants of the profound HL group were removed from the noisy environment and the study; however, no information is given concerning the continuation or not of noise exposure for the participants with mild, moderate, and severe HL (Hana & Bawi 2018). If during their treatment, and thus participation in the study, participants remained working in the same noisy environment, this could be considered as a confounding factor. Sun et al. (2020) excluded participants with other sources of HL. Nevertheless, no particular measures were taken to exclude participants with age-related or overall-noise-exposure-related cochlear damage. This fact, along with the small sample size, does not allow strong conclusions about the relation of ISSHL pathophysiological pathways and blood prestin level changes to be drawn.

Understandably, the aforementioned factors were better controlled in the rodent models. In the animal studies presented in this review, all animals were healthy, 6 to 20 weeks of age and with no prior exposure to ototoxic agents or noise before undergoing the experimental procedures (exposure to cisplatin, aminoglycosides, or noise). To confirm cochlear damage, rodents exposed to noise or other noxious agents underwent functional or histological testing. Degree of cochlear damage and HL was evaluated by means of ABR and DPOAEs, while three studies also included histological examination of the cochlea (Parham & Dyhrfjeld-Johnsen 2016; Dogan et al. 2018; Parham et al. 2019). In two of them, Parham et al. used the same methodology and focused on the mean loss of OHCs in each group as a function of normalized distance from the apex, using the total length of each histological specimen. In the context of the third study by Dogan et al., a pathologist blinded to the groups scored the specimens for their OHC count (number of OHCs with an intact nucleus) according to the four-point scoring system for cisplatin-induced ototoxicity defined by De Freitas et al. (2009). As a consequence, each deviation from baseline in the functional, histological, or prestin concentration outcomes can be safely attributed to the HL originating from experimental interventions.

Concluding, the discovery and validation of otologic biomarkers in human blood may be of great value to the prevention, early diagnosis, and prognosis of HL. To date, there is some evidence that prestin blood concentrations change in the case of acquired HL in rodents, and that this change is correlated with the degree of cochlear damage, the region of the cochlea that is affected, and the time interval between onset of disease and prestin measurement. These proof-of-concept studies provide important insight on the matter and provide preliminary evidence that prestin may indeed serve as a valuable biomarker for HL. However, larger-scale data are required to clarify the conditions under which blood prestin can be best used as a marker in the case of human subjects with SNHL. In human studies, specific methodological challenges have to be resolved before researchers are able to draw any conclusions. Future studies could be improved by larger samples, more detail on hearing phenotyping and clinical variables, prestin measurements at specific time points during the course of cochlear damage, clear segregation of the effect that temporary threshold shift has on prestin from the effect of permanent lesions, longer longitudinal experiments in unilateral and bilateral acquired HL, full audiometric profiling of subjects, detailed quantification of all factors that could have led to OHC damage, and definition of the clinical and genetic variability of each HL case.

## ACKNOWLEDGMENTS

E.I. conceptualized, designed the methodology, and executed the formal analysis, investigation and writing of the article. D.K. contributed to the investigation and writing of the original draft. K.P. contributed to the writing, review, and editing of the article. C.J.P. and A.B. supervised and contributed to the writing and editing of the article. All authors discussed the results and implications and commented on the article at all stages.

## Supplementary Material


