# Supplementary material for: Blood Prestin Levels in Normal Hearing and in Sensorineural Hearing Loss: A Scoping Review
Source: Ear Hear. 2021 Apr 22;42(5):1127–36. doi: 10.1097/AUD.0000000000001045 (PMC8378539; doi:10.1097/AUD.0000000000001045)
Supplement: Supplementary file 1 [file aud-42-1127-s001.pdf]

SM Table 1. Characteristics of included animal studies. All prestin blood level measurements were conducted by means of ELISA.

Author, Year, Journal	Title	Population	Summary	Audiological Assessment	Findings
Parham et al. *Hearing Research* 2019	Noise-induced trauma produces a temporal pattern of change in blood levels of the outer hair cell biomarker prestin	Male Wistar rats (6-9 weeks old)	Measurement of prestin blood levels at 4 h, 24 h, 48 h, 72 h, 7 and 14 days in 20 rats exposed to intense octave band noise for 2 h at either 110 or 120 dB SPL. Comparison with 26 naïve male rats.	Auditory brainstem responses (Tone-pips with 2 ms duration, 300 repetitions at a rate of 20/s, from 10 to 90 dB SPL with a 5 dB step, at 8, 16, and 24 kHz) & Distortion product otoacoustic emissions (80 /70 dB SPL, with an f2/f1 ratio of 1.2. At 4, 8, 16, 24, and 32 kHz) Day 0 & 14 [Histological evaluation of hair cell loss was held at day 14]	110 dB SPL group: Statistically non-significant, <5%, rise of prestin concentration at 4 hours post exposure, then a statistically significant gradual decrease to 10pg/ml (compared to baseline) at 14 days after exposure. 120 dB SPL group: 6 subjects presented increased prestin levels at 4 hours post exposure (22.8 ± 9 pg/mL) and 4 subjects decreased ones (17.3 ± 7 pg/mL). A statistically significant 10% decrease of prestin levels (compared to baseline) was

					observed at day 14 after exposure.
Dogan et al.<br><br>*American Journal of Otolaryngology*<br><br>2018	Utilizing prestin as a predictive marker for the early detection of outer hair cell damage	Male Wistar rats<br><br>(16–20 weeks old)	Measurement of prestin blood levels in 35 rats that received: 200 mg/kg/day of amicacin for (10 LAG), 600 mg/kg/day of amicacin for 10 days (6 HAG), one single dose of 5 mg/kg (9 LCIS), one single dose of 15 mg/kg (10 HCIS) and 10 controls.	Distortion product otoacoustic emissions (70/70 dB SPL, with an f2/f1 ratio of 1.22. at 2001, 3154, 4003, 6298 and 7998 Hz)<br><br>[Histological assessment of stria vascularis, organ of Corti, spiral ganglion according to 4-point scoring system for cisplatin-induced ototoxicity defined by Freitas et al.]	Dose-dependent cochlear damage and increase of prestin blood concentration.
Naples et al.<br><br>*Otology & Neurotology*<br><br>2018	Prestin as an Otologic Biomarker of<br><br>Cisplatin Ototoxicity in a Guinea Pig Model	Guinea pigs	Measurement of prestin blood levels performed at days 0, 1, 2, 3, 7, and 14 post-cisplatin<br><br>administration, in two groups of guinea pigs (one treated with diltiazem and one control)	Auditory brainstem responses<br>- tone bursts of 4, 8, 16, 24, and 32 kHz, 5-ms (2-ms rise/fall time), delivered at a rate of 21/s<br>- click-evoked	Rise in blood prestin levels (25.6%) at day 2 post cisplatin administration (precedes onset of significant ABR changes) in the control group.<br><br>No prestin concentration rise in the diltiazem group.

Liba et al.<br><br>*Otology & Neurotology*<br><br>2017	Changes in Blood Prestin Concentration After Exposure to Cisplatin	Mice<br><br>Guinea pigs	Measurement of prestin blood levels in 30 mice and 10 guinea pigs at 1, 3, 7, 14 days after 1 single dose of cisplatin at 8 mg/kg. (five mice were sacrificed at each time point, all guinea pigs were tested at each time point)	Auditory brainstem responses<br>- Clicks<br>- Rate of 21/s<br>- Step of 5 dB	Blood prestin concentrations rise, peak on days 7 (mice) and 3 (guinea pigs),<br><br>decline back to or below baseline / control levels 14 days after treatment.
Parham and Dyhrfjeld-Johnsen<br><br>*Otology & Neurotology*<br><br>2016	Outer Hair Cell Molecular Protein, Prestin, as a Blood Biomarker for Hearing Loss: Proof of Concept.	Male Wistar rats	Measurement of prestin blood levels in 21 rats of 6-11 weeks old at day 14 after intense octave band noise for 2 to 3 hours and six controls.	Auditory brainstem responses<br>- Tone-pips (5 ms duration at a rate of 21/s, 2 ms rise-fall time) at 8, 16, and 24 kHz from 90 dB SPL in steps of minimum 5 dB.<br>Distortion product otoacoustic emissions<br>- 70/70 dB SPL, f1:f2=1.2<br>- 4, 8, 16, 24, and 32 kHz	Noise-exposed rats demonstrated statistically significant<br><br>decrease in prestin concentrations 14 days post-exposure

LAG, low aminoglycoside group; HAG, high aminoglycoside group; HCIS, high cisplatin; LCIS, low cisplatin
